# Oat Bran Increased Fecal Butyrate and Prevented Gastrointestinal Symptoms in Patients With Quiescent Ulcerative Colitis—Randomized Controlled Trial

**DOI:** 10.1093/crocol/otaa005

**Published:** 2020-02-13

**Authors:** Margareta Nyman, Thao Duy Nguyen, Ola Wikman, Henrik Hjortswang, Claes Hallert

**Affiliations:** 1 Department of Food Technology, Engineering and Nutrition, Lund University, Lund, Sweden; 2 South Hospital, Stockholm, Sweden; 3 Department of Gastroenterology and Department of Clinical and Experimental Medicine, Linköping University, Linköping, Sweden; 4 Gastroenterology Unit at Norrköping Hospital, Norrköping, Sweden

**Keywords:** ulcerative colitis, β-glucan, oat bran, butyrate

## Abstract

**Background:**

Oat bran specifically increases colon butyrate concentrations and could therefore affect the progress of the disease in patients with ulcerative colitis (UC).

**Methods:**

Patients with UC in remission were enrolled in a controlled multicenter study and randomized to eat oat bran or low-fiber wheat products.

**Results:**

Ninety-four of the enrolled patients (*n* = 47 for both groups) completed the 24-week study. The oat bran group had significantly (*P <* 0.05) higher fecal butyrate concentrations and lower serum LDL levels, while deterioration of gastrointestinal symptoms was prevented, and subjective health maintained. The control diet significantly (*P <* 0.05) increased obstipation, reflux, and the symptom burden and had no effects on butyrate or LDL-cholesterol. The relapse rate was the same for both diets.

**Conclusions:**

Oat bran was well tolerated when given to patients with quiescent UC.

## Graphical Abstract

**Graphical_Abstract:**
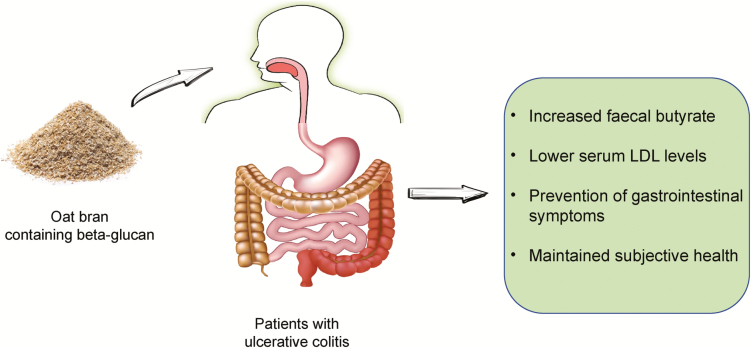

## INTRODUCTION

Ulcerative colitis (UC) is a chronic inflammatory disease affecting the colon and rectum with high variability between severe illness and periods of remission.^1^ The prevalence of UC is high in many Western societies, while it has been less frequent in Eastern Europe and Asia.^2,3^ During the last decades the disease has become more common also in these areas and it is rapidly increasing in developed Asian countries.^4^ Environmental factors, such as westernization and higher socioeconomic status, rather than genes, seem to trigger the disease, as supported by studies on monozygotic twins and the great geographical fluctuations in incidence rates of the disease.^5,6,7^ Several studies have suggested that the colon microbiota composition can be involved in the onset of the disease.^8^ In this respect, the diet composition and the type of dietary fiber consumed can be of importance for the progression of the disease. Dietary fiber is a heterogeneous group of substances with different functional characteristics and especially those fermented by the colon microbiota (prebiotics) may have an impact.^9^ The potential of fermentable fiber to alter the gut microbiota and increase the production of butyrate, a product lacking in the colon of patients with UC, may be important to maintain remission in patients with UC.^10–13^ Oats are of special interest, because the colonic formation of butyric acid increases specifically with this food item and is ascribed to its high content of β-glucan.^12,14^ Whole-grain oats have also been shown to provide a more favorable cecal microbiota composition in mice compared with a low-fiber diet, with a higher relative abundance of, for example, *Prevotella* and *Lactobacillus*.^15^

Butyric acid, the most important substrate for the colonic mucosa, decreases the intestinal permeability and consequently the passage of inflammatory substances from the colon into the blood may decrease. Patients with UC have a diminished capacity to utilize butyric acid, but the presence of increased amounts in the colon,^16^ for example, with enemas containing butyrate, may be helpful in this respect, as shown in some early short-term studies.^17,18^ Butyrate has been reported to inhibit the activity of many proinflammatory cytokines, but clinical studies are still scarce.^19,20^ The abundance of some butyrate-producing bacteria (eg, *Roseburia* and *Clostridium* clusters IV and XIVa) has been reported to be reduced in patients with UC compared with healthy controls,^10,21^ as well as the number of *Faecalibacterium prausnitzii* (also butyric acid producer) and *Eubacterium rectale.*^22^ To increase the formation of butyric acid in colon with specific fibers would therefore be of great interest. Results on effects of diet are often from prospective cohort studies with self-reported dietary intake with different types of questionnaires.^23,24^ Only weak associations between dietary fiber and the onset of UC are found in these investigations. In a recent review, it was also concluded that a high intake of FODMAP (fermentable dietary fiber of low molecular weight, such as oligosaccharides, disaccharides, monosaccharides, and polyols) even worsen the symptoms for patients with UC, which is against the fiber hypothesis.^25^ Consequently, there is a need for more prospective controlled trials to provide appropriate dietary recommendations.

The objective of this randomized controlled study in patients with quiescent UC was to examine whether large amounts of oat bran to the daily diet for 24 weeks have an impact on the fecal butyrate concentrations and the intestinal health, compared to a control group taking a low-fiber diet containing wheat. The intestinal health was evaluated by relapse rate, gastrointestinal symptoms, and subjective health. To our knowledge, we are the first to investigate the effect of a food item with specific properties on relapse-prone patients with UC in a long-term study with a large number of subjects.

## MATERIALS AND METHODS

### Patients

Relapse-prone patients with UC (*n* = 200), in clinical and endoscopic remission as determined with sigmoidoscopy and the SEO index less than 120,^26^ and regularly seen at 7 gastroenterology departments across central Sweden [Eksjö, Jönköping, Lidköping, Linköping, Norrköping, Skövde, and Stockholm (Södersjukhuset)], were invited to participate in the study. The recruitment lasted for 2 years.

Inclusion criteria for the study were adults with longstanding confirmed UC (>4 years) with stable medication (>6 months) and last clinical relapse within 15 months prior to entry. The sigmoidoscopy should be normal or near-normal with no signs of inflammation or friability, and clinical remission with a score less than 120 by the SEO index.^26^ The colitis was evaluated with sigmoidoscopy using the Mayo endoscopic score, ranging from 0 to 3 according to Schroeder et al.^27^ Exclusion criteria were assumed poor compliance to study protocol and topical treatment with 5-ASA or immunomodulators, steroid medication, and antibiotics.

Relapse was identified as an initial recent bloody diarrhea supported by endoscopy, showing light bleeding mucosa and an overall diagnosis by the responsible gastroenterologist. The follow-up of relapse occurred outside the study.

### Study Design and Diet Supplements

The patients were randomized to either the active group adding 60 g of oat bran corresponding to an intake of 12 g dietary fiber (consistent to 6 g of β-glucan) to the daily diet, or the control group adding low-fiber wheat products (providing 5 g dietary fiber daily and <0.5 g β-glucan) for 24 weeks, while ongoing drug treatment was unchanged. This means that the patients from the oat group increased their daily dietary fiber intake considerably (with about 60%) compared with the average dietary fiber intake for adults in Sweden (17 and 20 g/day for women and men, respectively) according to the National Food Agency. Both sets of diet supplements comprised a selection of biscuits, porridge, or breakfast cereals that were handed out blinded by study nurses, notifying diet compliance and clinical events during the trial including, for example, start on antibiotics. The intake of dietary fiber at baseline was also assessed by the study nurse and to be within reported national data. All patients were instructed to undergo sigmoidoscopy within 3 working days at signs of relapse.^26,27^ Endpoint was set at week 24 or confirmed clinical and endoscopic relapse, which was the primary outcome of the study.

### Study Evaluation

The primary outcome of the study was confirmed by clinical and endoscopic relapse as defined above, that is, as greater than 120 with the SEO index or with 2 (moderate) or 3 (severe) with the Mayo endoscopic score.

The secondary outcomes included analyses of short-chain fatty acids (SCFAs), including butyric acid, and registration of gastrointestinal symptoms and subjective health. SEO index, serum lipids, and blood glucose were also analyzed.

Short-chain fatty acids were analyzed in feces every fourth week (0, 4, 8, 12, 16, 20, and 24 weeks) and in serum (not systematically collected from all patients and times). Analyses of fasting serum lipids, and blood glucose were taken bimonthly (0, 8, 16, and 24 weeks). All patients were seen monthly by a nurse at each hospital and this person was also handing out the diet supplements and collecting frozen stool samples. The stool and blood samples were saved in the freezer until analysis.

Gastrointestinal symptoms were measured bimonthly (0, 8, 16, and 24 weeks) by the Gastrointestinal Symptom Rating Scale (GSRS), a self-administrated questionnaire consisting of 15 items designed to assess 5 gastrointestinal syndromes (indigestion, diarrhea, constipation, abdominal pain, and gastroesophageal reflux).

Subjective health was measured with the Short Health Scale (SHS), a self-administered questionnaire containing 4 questions related to major health dimensions in inflammatory bowel disease.^28^ The symptoms reported for subjective health were symptom burden (SHS1), impairment of daily functions caused by the disease (SHS2), disease-related worries (SHS3), and general well-being (SHS4). The responses were graded on a 100 mm Visual Analog Scale, where higher values indicate worse subjective health and decreased quality of life. The results are presented as an individual score for each dimension.

The SEO index, a noninvasive measure of remission based on stool frequency, blood in stools, and levels of hemoglobin, albumin, and Westergren erythrocyte sedimentation rate were also registered.^26^ Index scores less than 120 indicate clinical remission.

Compliance was graded 1–4 on 6 separate visits, recorded by the study nurses, where 1 was reported as full adherence to protocol.

### Analyses

Fecal SCFAs were determined using gas chromatography, involving water extraction of the SCFAs and direct injection procedure allowing measurement of 8 SCFAs, including acetic-, propionic-, and butyric acids.^29^ This means that 1316 × 2 (*n* = 2632) SCFA analyses were performed. However, only results on SCFAs in week 0 and 24 are given in Table 2, because only these analyses were significant.

**TABLE 1.** Baseline Characteristics of Patients With Ulcerative Colitis Randomized to Take Oat Bran (*n* = 67) or a Control Diet Containing Wheat (*n* = 63)

Treatment	Oat Bran (*n* = 67)	Control (*n* = 63)
Gender, male (%)	46	43
Ethnicity, white (%)	100	100
Smoker (%)	12	10
Ex-smoker (%)	17	17
Disease duration (median years)	11	11
5-ASA (%)	97	98
Extent of disease (%)		
Proctitis	22	23
Distal colitis	56	53
Total colitis	22	24
Indigestiona	1.09 ± 0.07 (*P* < 0.05)	0.88 ± 0.08
Diarrheaa	0.63 ± 0.09	0.54 ± 0.09
Obstipationa	0.37 ± 0.06	0.46 ± 0.08
Abdominal paina	0.63 ± 0.07 (*P* < 0.05)	0.46 ± 0.07
Refluxa	0.37 ± 0.08	0.32 ± 0.07
Total gastrointestinal symptomsa	0.62 ± 0.04 (*P* = 0.0867)	0.53 ± 0.05
Symptom burden (SHS1)a	0.79 ± 0.19	0.58 ± 0.17
Daily function (SHS2)a	0.41 ± 0.14	0.31 ± 0.13
Disease-related worry (SHS3)a	0.57 ± 0.18	0.62 ± 0.20
General well-being (SHS4)a	1.39 ± 0.27	1.06 ± 0.24
SEOa	102.2 ± 2.1	101.2 ± 1.5
LDL (mmol/L)^a^	2.86 ± 0.13	2.98 ± 0.11
Fasting blood sugar (mmol/L)^a^	5.18 ± 0.20	4.90 ± 0.07

^a^Mean ± SEM, when means are different between groups *P*-values are included.

**TABLE 2.** Changes in Fecal (*n* = 94) and Serum (*n* = 14) SCFA Concentrations in Patients with UC, Eating Products Containing Oat Bran (12 g Dietary Fiber, 6 g β-Glucan) (*n* = 47) or Wheat (Control, 5 g Dietary Fiber, <0.5 g β-Glucan) (*n* = 47), and Fulfilling the 24-Week Study (*n* = 47/47) (Mean ± SD)

	Oat Bran (12 g Dietary Fiber)		Wheat (5 g Dietary Fiber)	
SCFAs	Week 0	Week 24	Week 0	Week 24
Total in feces (µmol/g)	91 ± 8	102 ± 8*	77 ± 7	83 ± 7
Acetic acid	53 ± 5	57 ± 5	45 ± 4	52 ± 5
Propionic acid	16 ± 2	18 ± 2^**^	13 ± 1	13 ± 2
Isobutyric acid	2.3 ± 0.3	2.2 ± 0.2^(*)^	1.9 ± 0.2	1.7 ± 0.2
Butyric acid	14 ± 2	18 ± 2^*,¶^	11 ± 1	11 ± 1
Isovaleric acid	3.2 ± 0.4	3.4 ± 0.3	2.7 ± 0.3	2.7 ± 0.3
Valeric acid	2.1 ± 0.3	2.4 ± 0.3*	1.6 ± 0.2	1.7 ± 0.2
Caproic acid	0.8 ± 0.2	1.1 ± 0.2^¶^	0.7 ± 0.2	0.8 ± 0.2
Heptanoic acid	0.08 ± 0.03	0.14 ± 0.04^(¶)^	0.11 ± 0.04	0.08 ± 0.03
Total in blood (µmol/L)	218 ± 15	241 ± 25	248 ± 22	222 ± 10
Acetic acid	155 ± 10	171 ± 17	178 ± 20	154 ± 5
Propionic acid	10 ± 0	13 ± 1	12 ± 1	11 ± 2
Isobutyric acid	14 ± 1	15 ± 2	16 ± 1	15 ± 1
Butyric acid	0.3 ± 0.2	0.1 ± 0.0	0.2 ± 0.1	0.1 ± 0.1
Isovaleric acid	38 ± 5	41 ± 7	42 ± 4	42 ± 5

Significant differences between oat bran and wheat at week 0 or at week 24: (*)*P* = 0.05–0.1, *P < 0.05, **P < 0.01.

Values at week 24 are significantly different from values at week 0: (^¶^)*P* = 0.05–0.1, ^¶^*P* < 0.05.

SCFAs in serum were determined by protonating the SCFAs with hydrochloric acid.^30^ A hollow fiber for supported liquid membrane extraction was immersed in the serum solution to extract and enrich the SCFAs. After extraction, the SCFAs were flushed from the fiber lumen and mixed with 2-ethylbutyric acid (internal standard) before analyzed with gas chromatography by injecting the samples on a fused-silica capillary column. SCFAs in serum were determined in 14 subjects during the 7 time points 196 × 2 (*n* = 392).

LDL cholesterol in serum was determined using kits purchased from Thermo Scientific (Middletown, USA) and fasting blood glucose by kits from HemoCue B-glucose (HemoCue AB, Ängelholm, Sweden).

### Calculations and Statistical Analyses

**FIGURE 1.**
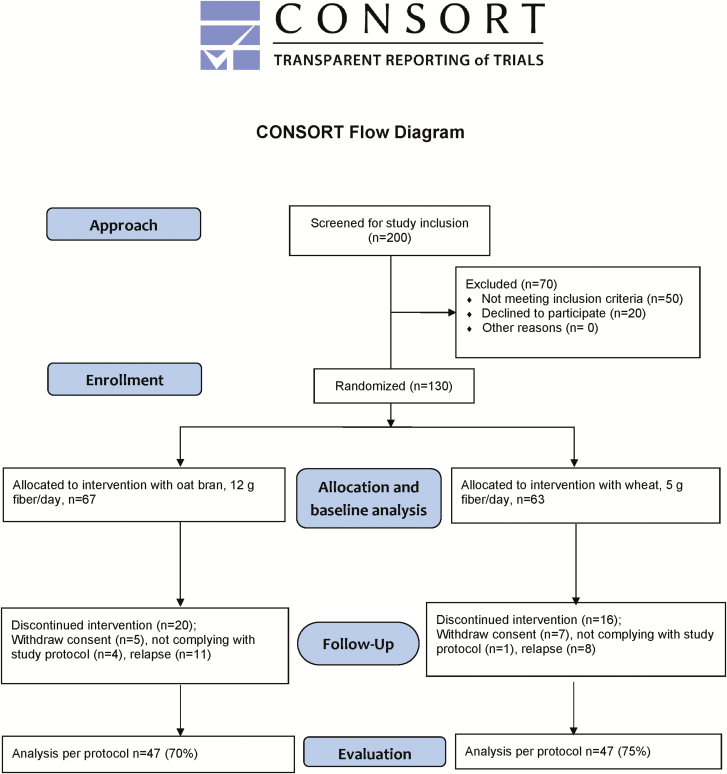
Consort flow diagram.

The primary outcome was evaluated according to intention to treat, that is, the possibility to decrease the relapse rate with oat bran. Significances between the numbers of relapses with the 2 diets were evaluated by a χ ^2^ test. Results from secondary outcomes were analyzed per protocol, that is, on patients who did not discontinue the treatment according to Figure 1. Two comparisons were then performed and in one of these the 2 diet groups were compared at the different time points. This means that the mean values of all patients remaining in the study were compared at inclusion (*n* = 67/63) and after 8 (*n* = 57/53), 16 (*n* = 51/50), and 24 (*n* = 47/47) weeks and these values are presented in Figure 3. SCFAs were evaluated in the same way, but only data at inclusion and after 24 weeks are given in Table 2. Differences between groups were determined by nonparametric unpaired, 2-tailed Mann–Whitney test (GraphPad Prism 7.04). To get a more normal distribution of the SCFA values, these were also logarithmic transformed, but no differences could be seen whether these were logarithmic transformed or not.

The other comparison was an intragroup comparison of GSRS, SHS, SCFAs, and SEO over time, that is, the effect of the intervention was studied. This means that values between baseline and the specific time points were compared along the 24 weeks of intervention for each person eating one of the 2 diets, but for obvious reasons only for those who participated in the entire study (*n* = 47/47). Nonparametric Wilcoxon signed ranking test was used in this case (Tables 2 and 3).

**TABLE 3.** Changes in Gastrointestinal Symptoms, Subjective Health, SEO Index, LDL Cholesterol, and Fasting Glucose Over Time in Patients With Ulcerative Colitis, Eating Products Containing Oat Bran (12 g Dietary Fiber, 6 g β-Glucan) or Wheat (Control, 5 g Dietary Fiber, <0.5 g β-Glucan), and Fulfilling the 24-Week Study (*n* = 47/47) (Mean ± SD^a^)

Analyses	0 Weeks	8 Weeks	16 Weeks	24 Weeks
Gastrointestinal symptoms				
*Indigestion*				
Oat bran	1.11 ± 0.58	1.10 ± 0.64	0.94 ± 0.61*	1.07 ± 0.64
Control	0.86 ± 0.61	0.92 ± 0.70	0.86 ± 0.59	0.99 ± 0.66
*Diarrhea*				
Oat bran	0.72± 0.77	0.81 ± 0.99	0.71 ± 0.70	0.80 ± 0.93
Control	0.45 ± 0.56	0.41 ± 0.59	0.40 ± 0.57	0.58 ± 0.84
*Obstipation*				
Oat bran	0.38 ± 0.48	0.54 ± 0.81	0.41 ± 0.44	0.42 ± 0.45
Control	0.42 ± 0.58	0.61 ± 0.90(*)	0.50 ± 0.77	0.60 ± 0.74*
*Abdominal pain*				
Oat bran	0.62 ± 0.54	0.67 ± 0.76	0.50 ± 0.46	0.60 ± 0.51
Control	0.39 ± 0.47	0.67 ± 0.66^***^	0.43 ± 0.49	0.59 ± 0.6*
*Reflux*				
Oat bran	0.39 ± 0.61	0.36 ± 0.55	0.34 ± 0.65	0.44 ± 0.63
Control	0.23 ± 0.48	0.28 ± 0.52	0.28 ± 0.51	0.41 ± 0.61* ^,¶,ǂ^
*Total gastrointestinal symptoms*				
Oat bran	0.64 ± 0.38	0.69 ± 0.49	0.58 ± 0.37	0.67 ± 0.41
Control	0.47 ± 0.35	0.58 ± 0.45*	0.49 ± 0.36	0.64 ± 0.49* ^,ǂ^
Subjective health				
*Symptom burden (SHS1)*				
Oat bran	0.9 ± 1.6	1.2 ± 1.9	0.9 ± 1.5	1.3 ± 2.0
Control	0.6 ± 1.5	0.7 ± 1.9	0.7 ± 1.6	1.3 ± 2.1^*,¶^
*Daily function (SHS2)*				
Oat bran	0.4 ± 1.0	0.3 ± 0.9	0.3 ± 0.8	0.6 ± 1.5
Control	0.4 ± 1.2	0.4 ± 1.6	0.2 ± 0.6	0.7 ± 1.7
*Disease-related worry (SHS3)*				
Oat bran	0.5 ± 1.4	0.6 ± 1.5	0.4 ± 1.1	0.6 ± 1.3
Control	0.7 ± 1.8	0.5 ± 1.7 (*)	0.8 ± 2.2	0.9 ± 1.6*
*General well-being (SHS4)*				
Oat bran	1.1 ± 1.8	1.7 ± 2.3	1.2 ± 1.8	1.3 ± 1.6
Control	1.0 ± 1.7	1.8 ± 2.6	1.4 ± 2.2	1.4 ± 2.0
*SEO*				
Oat bran	102.6 ± 16.8	105.4 ± 19.5	102 ± 17	105.5 ± 21.9
Control	102 ± 12.2	101.2 ± 10.9	104.4 ± 18.7	102.3 ± 19.3
LDL cholesterol (mmol/L)				
Oat bran	2.9 ± 1.0	2.6 ± 0.8(*)	2.7 ± 0.9	2.6 ± 1.0*
Control	3.0 ± 0.9	3.0 ± 1.0	2.9 ±1.0	2.9 ± 0.8
Blood sugar (mmol/L)				
Oat bran	5.0 ± 1.6	5.2 ± 1.3	5.3 ± 1.0	5.1 ± 1.0
Control	4.9 ± 0.6	5.6 ± 2.9^***^	5.1± 0.7(*)	5.2 ± 0.6^**^

^a^Significant differences for each diet group compared with week 0. (*) *P* = 0.05–0.1, * *P* < 0.05, ** *P* < 0.01, *** *P* < 0.001.

With week 8 ¶ *P* < 0.05; with week 16, ǂ *P* < 0.05.

To get a better overview of the abundant variables from the 2 diets evaluated in the study, relationships between parameters were identified and compared after finished treatment (week 24) by using Projections to Latent Structures—Discriminant Analysis (PLS-DA) with SIMCA software (version 15, Umetrics, Umeå, Sweden). PLS-DA is a multivariate statistical method, enabling evaluation of complex matrices of data information and visualizing trends. By removing noise from the underlying “true” information, it is possible to classify observations into appropriate groups and identify relationships between objects. Behind each variable (4-point star in Figure 4), results from all the subjects (*n* = 94) in the study are hidden. The markers for each of the 2 diets contain results on all variables collected from one of the 2 groups (*n* = 47). In this way it was possible to visualize associations between diets and analyzed data per protocol at week 24. Spearman test was used to identify correlations between different variables (Supplementary Table S1).

## ETHICAL CONSIDERATIONS

The Ethical Committee at Linköping University approved the investigation (Linköping, Sweden) (No. M19-04). The trial was registered at Eudra No. ISRCTN75453816.

## RESULTS

### Patient Characteristics

Fifty of the 200 approached subjects did not meet the study criteria and further 20 declined to participate (Fig. 1). The remaining 130 patients (72 women) aged 18–78 years (median years 45) with a median disease duration of 11 years entered the trial. Of them 23% had total colitis.

Of the subjects enrolled in the study, 67 were randomized to take oat bran and 63 to take the control diet (Fig. 1). The baseline characteristics of these patients are given in Table 1. After randomization there were some dropouts (*n* = 17), corresponding to 9 (13%) and 8 (13%) from the groups given oat bran and the control, respectively. Twelve of these subjects withdrew their consent after a couple of weeks [*n* = 5 and *n* = 7 for the oat bran (7%) and control group (11%), respectively], and further 5 did not comply with the study protocol [*n* = 4 and *n* = 1 for the oat bran (6%) and control group (2%), respectively]. Nineteen patients were withdrawn because of a relapse during the study (see Primary Outcome). Consequently, 94 patients (47 from both groups) completed the entire study, corresponding to 70% and 75% in the groups given oat bran and the control diet, respectively. These were analyzed per protocol.

The compliance rates were similar with both diets, 81% and 83% for the oat bran and control group, respectively.

### Primary Outcome

**FIGURE 2.**
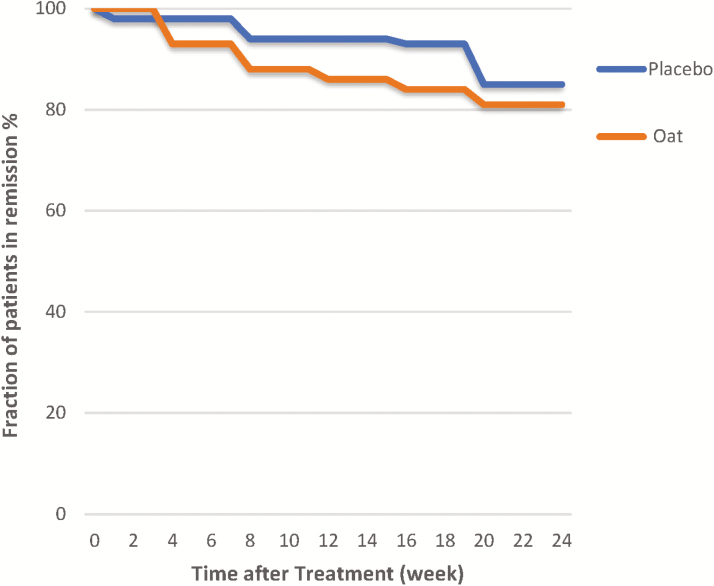
Kaplan–Meier curve assessing the influence of diet—oat bran or control (wheat)—on clinical relapse rate in patients with ulcerative colitis.

There was no significant difference in the relapse rate between the 2 groups (Fig. 2). After the 24-week study, 19 patients (*n* = 11 and *n* = 8 for the oat bran and control group, respectively) had a relapse. The distribution between men and women was equal. In the group eating oat bran 4 relapses occurred already before 4 weeks, 3 between 4 and 8 weeks, and the remaining 4 between 12 and 20 weeks, corresponding to 81% relapse-free patients at the end of the study. In the control group, one had a relapse 1 week after randomization, additional 2 before 8 weeks, 1 before 16 weeks, and 4 during the last 4 weeks (ie, at 20 weeks), which corresponded to 85% relapse-free patients when the study was finished. No differences could be seen in the SEO index between groups at any time point, neither at baseline nor along the intervention period.

### Secondary Outcomes

#### Fecal SCFAs

There were no significant differences in fecal SCFAs between groups at the entry of the study (week 0) but the individual variation was quite high in both groups (Table 2). After the 24-week intervention, subjects eating the oat bran products had significantly higher fecal concentrations than those eating the control concerning total SCFAs (102 vs 83, *P* < 0.05), butyric acid (18 vs 11, *P* < 0.05), propionic acid (18 vs 13, *P* < 0.01), and valeric acid (2.4 vs 1.7, *P* < 0.05). Also isobutyric acid tended to be higher with oat bran after the intervention (2.2 vs 1.7, *P* < 0.1).

After the 24-week intervention period with oat bran, these subjects had a significantly (*P* = 0.029) higher fecal concentration of butyric acid (18 µmol/g) compared with values at entry (14 µmol/g) (Table 2), which corresponded to an increase of about 30%. Furthermore, the concentration of the minor SCFA caproic acid was also significantly higher (1.1 vs 0.8 µmol/g, *P* = 0.046) and that of heptanoic acid tended to be higher (0.14 vs 0.10 µmol/g, *P* = 0.059). No significant differences could be seen in subjects eating the control diet with low amounts of fiber in any of the SCFAs analyzed during the intervention period.

#### Serum SCFAs

Analyses of SCFA were also performed in blood in some patients and these were evaluated by a discriminant analysis over time (week 0, 4, 8, 12, 16, and 24) (Table 2). It could then be seen that the concentrations of acetic-, propionic-, and butyric acids in serum of subjects’ eating oat bran increased in more subjects than those eating control diet. Concerning propionic acid, it increased in all subjects investigated, while only 1 subject had increased concentration in the control group.

#### Serum cholesterol

**FIGURE 3.**
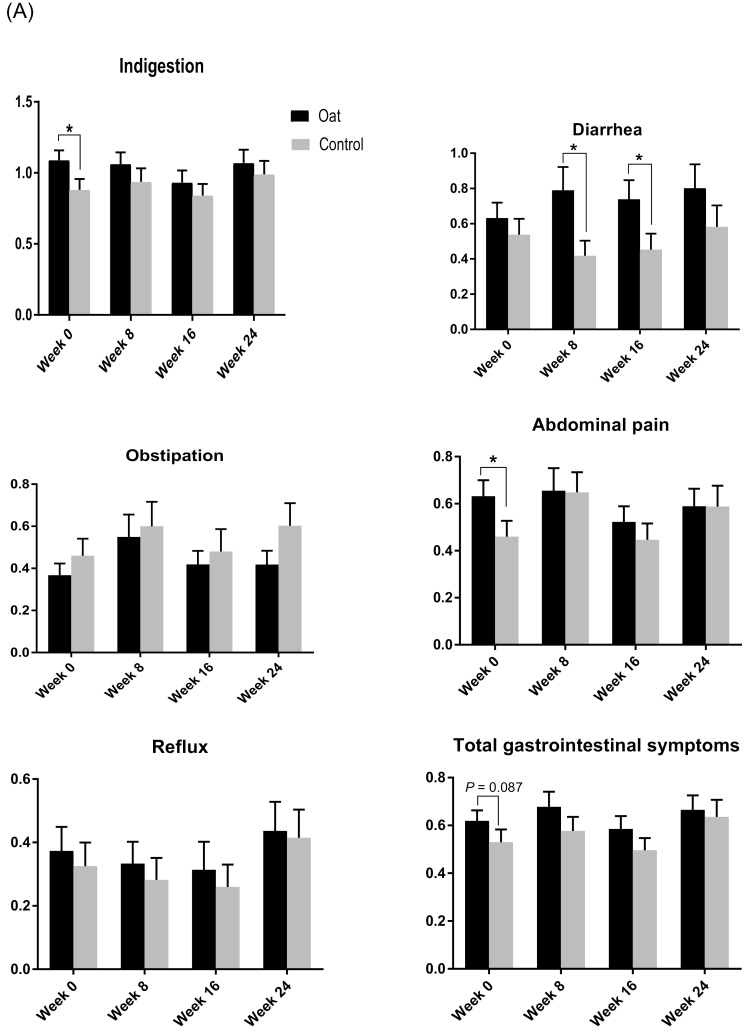

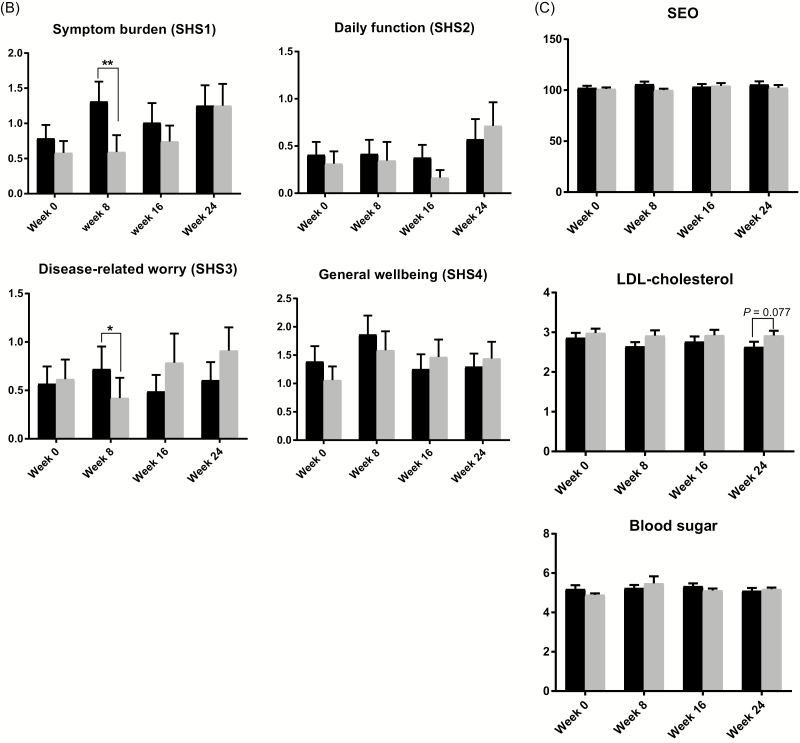
Comparison of (A) gastrointestinal symptoms, (B) subjective health, (C) SEO index, LDL cholesterol, fasting glucose, and compliance in patients with ulcerative colitis eating products containing oat bran or a control diet (wheat) at start (week 0, *n* = 67/63) and after 8 (57/53), 16 (51/50), and 24 weeks (47/47); mean ± SEM.

The levels of LDL cholesterol in the oat bran group tended to be lower than in the control group (2.6 vs 2.9 mmol/L, *P* = 0.077) at week 24 (Fig. 3). No other significant differences could be seen between groups at different time points.

The LDL cholesterol levels were lower after the 24-week intervention period with oat bran (2.6 mmol/L) than at inclusion (2.9 mmol/L, *P* < 0.05) (Table 3) and tended (*P <* 0.077) to be lower already after 8 weeks. There were no differences in LDL cholesterol levels during the intervention period with the control diet.

#### Blood glucose

There were no differences in blood glucose levels between groups at any time point (Fig. 3).

Blood glucose levels were similar to the oat bran group over the 24-week intervention (Table 3). Subjects in the control group had higher blood glucose levels after 8 and 24 weeks (5.6 and 5.2 mmol/L, respectively, *P* < 0.01 to 0.001) compared with values at entry (4.9 mmol/L). The blood glucose values also tended to be higher at week 16 (*P* = 0.078).

#### Gastrointestinal symptoms (GSRS)

Baseline characteristics were higher in the group randomized to eat oat bran (*n* = 67) for indigestion (1.09 vs 0.88, *P* < 0.05) and abdominal pain (0.63 vs 0.46, *P* < 0.05) compared with the group randomized to eat the control diet (*n* = 63) (Table 1). No other differences in the GSRS dimensions (ie, diarrhea, constipation, and gastroesophageal reflux) between groups were seen after randomization, except that total GSRS scores tended to be higher (0.62 vs 0.53, *P* = 0.087) for the group randomized to eat oat bran than the group randomized to eat the control diet.

The higher baseline levels of indigestion, abdominal pain, and total GSRS score in the oat bran group were leveled out to a great extent over time (Fig. 3). However, diarrhea was higher in the group eating oat bran compared with the control group at weeks 8 and 16 (0.79 vs 0.42, *P* < 0.05 and 0.74 vs 0.45, *P* < 0.05, respectively). At the end of the study, there were no significant differences between diet groups.

When studying the effect of the intervention, that is, the changes over time from week 0 to week 24 for each person eating respective diet, the gastrointestinal symptoms for the oat bran group were kept quite similar as at inclusion, except for a lower value of indigestion after 16 weeks (0.94 vs 1.11 at inclusion, *P* < 0.05) (Table 3). On the other hand, in the control group the values increased over time concerning obstipation (0.60 vs 0.42 at inclusion, *P* < 0.05) and abdominal pain (0.59 vs 0.39 at inclusion, *P* < 0.05). The values for reflux were also higher at the end of the study compared with those at week 0 (0.41 vs 0.23 at inclusion, *P <* 0.05) and at 8 and 16 weeks (0.41 vs 0.28, *P* < 0.05). Furthermore, total GSRS scores were higher at the end of the study compared with week 0 (0.64 vs 0.47, *P* < 0.05) and 16 weeks (0.64 vs 0.49, *P* < 0.05). The GSRS values were higher already at 8 weeks (0.58 vs 0.47 at week 0, *P* < 0.05).

#### Subjective health

There were no differences at baseline for the different dimensions of subjective health between the diet groups (Table 1).

Higher values of symptom burden (SHS1, 1.3 vs 0.6, *P* < 0.01) and disease-related worries (SHS3) (*P* < 0.05) were seen in the oat bran group at week 8 compared with the control group (Fig. 3). No other differences could be seen between groups at the specific time points.

No significant differences were seen in scores for subjective health (SHS1–SHS4) over time for the group eating oat bran, while there was an increase in symptom burden (from 0.6 to 1.3, *P* < 0.05) and disease-related worries (from 0.7 to 0.9, *P* < 0.05) after 24 weeks in the control group (Table 3).

### PLS-DA Analysis

**FIGURE 4.**
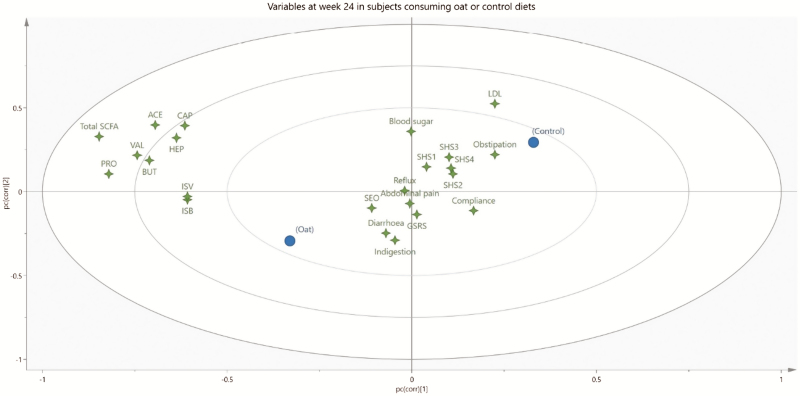
PLS-DA loading plot displaying relation of variables in patients with ulcerative colitis receiving oat bran (with high amounts of dietary fiber and β-glucan) (*n* = 47) or control (wheat with low amounts of dietary fiber) (*n* = 47) after the 24-week intervention. The 2 diets (oat and control, respectively) are shown as filled circles (*n* = 47 for each diet), while gastrointestinal symptoms (GSRS) (Indigestion, Diarrhea, Obstipation, Abdominal pain, Reflux, and GSRS—total symptoms), subjective health (SHS1—symptom burden, SHS2—daily function, SHS3—disease-related worry, and SHS4—general well-being), SEO, fecal SCFAs (total SCFAs, ACE—acetic acid, PRO—propionic acid, BUT—butyric acid, ISB—isobutyric acid, ISV—isovaleric acid, HEP—heptanoic acid, CAP—caproic acid), LDL cholesterol (LDL), blood sugars, and compliance are shown as 4-point stars (*n* = 94 for each 4-point star).

The relation between the 2 diets and all analyzed parameters from week 24 are visualized in Figure 4. Behind each star there are results from 94 individuals, and behind the circles, which represent the 2 diet groups, all values evaluated from the oat bran and the control groups are summarized. Oat bran, to the left in the figure, is associated with higher amounts of fecal SCFAs (all values clustered together to the left) and lower LDL values (located to the right). The circle for the control group eating wheat is placed to the right in the figure, that is, in the opposite direction to the oat bran group. This group is associated with the higher values of obstipation and LDL and to some extent also poorer perceived subjective health, that is, daily function (SHS2), disease-related worries (SHS3), and general well-being (SHS4). On the other hand, compliance is better with the control diet.

Figure 4 also shows that the dimensions of subjective health were interdependent and formed one cluster in the plot, while the SCFAs formed another cluster in the reverse direction to the subjective health. The dimensions of gastrointestinal symptoms were more independent and divergent. Diarrhea, for example, was located quite far from constipation, which could be expected. In the middle of these 2 dimensions, abdominal pain and indigestion were placed.

Some correlation numbers were calculated on some of the variables evaluated. Indigestion was linked with subjective health (SHS1–4), obstipation, reflux, and abdominal pain (Spearman coefficient *r* = 0.67) and all these correlations were significant. Furthermore, there tended to be a weak negative correlation between fecal butyric acid and SHS3 (disease-related worry, *P* = 0.100, *r* = −0.175), and the higher the concentration of butyric acid, the lower the values of SHS3. Similarly, there was also a weak negative correlation between fecal acetic acid and SHS3 and SHS4 (general well-being and obstipation, respectively) (*r* = −0.185 to −0.189).

### Dropout Patients

Thirty-six patients dropped out from the study some of them quite early, that is, 10 before/at 8 weeks. Baseline characteristics for the dropout patients were compared with those who fulfilled the study, where the aim was to see if any parameters registered at the beginning of the study could give an indication of future health conditions for these patients. It was then shown that the scores for compliance were much lower for the dropout patients compared with those who fulfilled the study (*P <* 0.0001). Furthermore, the initial values for general well-being tended to be lower compared with those who completed the study (*P =* 0.108). The other parameters were similar.

## DISCUSSION

In the past decades, there has been a growing interest in the health effects of butyric acid, due to its capacity to improve colon homeostasis, anti-inflammatory properties, and modulation of intestinal permeability.^31^ This is especially interesting in subjects with UC, who have a diminished capacity to utilize butyric acid.^16^ For this reason, a number of experiments have been carried out in subjects with UC with enemas containing butyrate to increase the presence of this bacterial metabolite in colon.^20,32–34^ However, results are not clear, and the ambiguous results may be due to several reasons, such as the short duration of many studies, too small numbers of subjects examined, different types of colitis investigated, different concentrations of butyric acid delivered, and perhaps also difficulties to take enemas.^20^ A better way could be to supply the butyric acid via the diet and utilize colon fermentation of dietary fiber. The present study is unique, because it is studying the effect of a fiber-rich food item, known to generate high amounts of specifically butyric acid, and with a large number of subjects for a comparatively long time.

An important result from the study is that patients with UC in remission may take large amounts of oat bran to increase fecal butyrate levels, with no excess risk of relapse and at the same time keep the subjective health and as an additive effect also decrease LDL cholesterol levels. This contrasts with wheat, which correlated with more pronounced gastrointestinal symptoms and increased values of obstipation, abdominal pain, and reflux as well as of higher scores of symptom burden and with no increase in SCFA concentrations. The most noteworthy is that the oat bran diet increased fecal butyrate levels, the reason behind attenuated mucosal inflammation and less number of lesions, observed in some studies of patients with UC given butyrate enemas.^17–20^ Butyrate enemas have also been reported to decrease oxidative stress in healthy subjects, as judged by the increased colonic levels of glutathione, another factor of interest in relation to UC and its origin.^35^

The risk for relapse in patients with UC, without optimizing the therapy can be as high as 30%–40% per year, implying considerable morbidity among people within working age.^36,37^ The possibility to increase the concentration of butyric acid with the diet is therefore interesting, but dietary recommendations are scarce and a diet low in dietary fiber is often prescribed. Although the etiology of UC is multifactorial, a hypothesis gaining increasing attraction implicates impaired epithelial barrier function leading to alleviated inflammation. In this respect, dietary fibers giving high amounts of butyric acid during microbial degradation in the colon are of great interest, because butyric acid is the most important substrate for the colonocytes. Notably, a daily intake of oat bran increased the fecal concentration of butyric acid significantly from 14 to 18 µmol/g, that is, approximately 30%. No such increase could be seen with wheat. It can be argued that the relapse rate was the same in both groups. However, it may take a longer time to see the difference in the relapse rate between diets. Furthermore, the relapse rate was quite low with both diets.

Another strength of this study is the low rate of dropouts and that it was not higher in the group eating oat bran. Thus, 70% of the subjects included in the oat bran group fulfilled the study, indicating that the high-fiber diet was almost as tolerated by the subjects as the low-fiber diet (75%). Usually a high-fiber intake is associated with lower compliance also among healthy people.^38^ Interestingly, adding large amounts of oat bran to the diet brought no increase in abdominal discomfort or reason for leaving the study. Only occasional study patients could identify the oat bran supplement, 3 of whom being farmers, indicating that it is possible to make tasty products with high-fiber materials.

The major weakness comprises the limited sample size of feces collected and the comparatively short duration of the trial, although long compared with most other studies. Fecal sampling is complicated, because it is difficult to standardize between subjects and between different times. This was also shown by a quite high variation between the 2 samples collected by each subject every time point. Another pitfall is the quite different levels of SCFAs seen in different subjects. Some subjects have low amounts of SCFAs in themselves, whereas others have quite high amounts. However, although the levels vary considerably between subjects, there was an increase in butyric acid with the oat fiber diet during the intervention period, which contrasted with the wheat diet containing low amounts of fiber (12 g vs 5 g/day), which is a strength. Furthermore, if the study had lasted for a longer time the differences might have been even greater.

Another weakness is that most SCFAs formed in the colon are utilized by the mucosa/absorbed into the blood and only smaller amounts are excreted in feces (5%). Thus, it can be questioned if the fecal concentrations of SCFAs are reflecting events in colon. However, since UC often occurs in the distal part of the colon (in this study close to 80%), analyses in feces would reflect the relative concentration in the distal part of the colon and thus be a relevant measure. On the other hand, since most SCFAs are absorbed, another way to evaluate the effects of diet in subjects with UC could be to analyze SCFAs in blood. Such analyses are also much easier to standardize.^39^ SCFAs in the serum of the subjects in the present study were also measured. Although quite a few serum samples, the effect of the oat fiber diet was reflected in serum, with higher levels of propionic acid with the oat bran diet than the control. This should be considered when designing experiments in the future.

There are not that many studies on effects of diet on UC, but some show beneficial effects with cereal dietary fiber.^11–13^ In a review it was however concluded that a high intake of FODMAP, that is, dietary fiber components giving high amounts of SCFAs, was of major concern for subjects with UC.^25^ Concerning this, it is important to keep in mind that most dietary fibers of low molecular weight and high solubility are rapidly fermented in the proximal colon, while colonic diseases frequently occur in the distal part of colon. The reason for the severe gastrointestinal symptoms in subjects with UC may instead be due that these types of low-molecular-weight dietary fiber are quite resistant to fermentation in these subjects (due to another microbiota), and therefore there is a considerable accumulation of water in the gut. A similar phenomenon was seen in a study of subjects with irritable bowel syndrome.^40^ They had severe abdominal pain, borborygmi, and bloating after intake of lactulose, which could be correlated to significantly lower serum SCFA concentrations in these subjects compared to healthy controls.^40^ A possibility to correct for this inborn “error” is to mix rapidly fermentable dietary fibers with more slowly fermentable ones, that is, fiber polysaccharides with a high degree of polymerization and low solubility together with dietary fiber of high solubility and/or low molecular weight, and in this way shift the SCFA formation from the proximal to the distal part of colon, as seen in rats.^41^ The type of oat fiber in this study was rather insoluble with large amounts of fiber polysaccharides of high molecular weight and thus slowly fermented preferably in the distal part of the colon.

In the present study, the levels of butyric acid increased first after 24 weeks with oats, whereas there was an increase already after 4 weeks in a previous study on patients with UC consuming β-glucan-enriched oat.^12^ This may be due to the comparatively lower amount of β-glucan consumed daily in this study (6 g vs 10 g in the previous study). Another difference was the lower number of butyrate responders in the present study, 80% versus 90% in the previous study. Otherwise, the mean fecal butyrate levels were in the same range in the 2 studies and also compared with a study on healthy subjects consuming β-glucan-enriched oats.^42^ However, in the study on healthy subjects, it took longer times, 8 weeks, to see an effect of the used oats.

The cholesterol levels were lower in the group who added oat bran to the diet for 24 weeks than the control group, eating wheat. This agrees with other studies^43^ and is consistent with the food and drug administration who has stipulated that the intake of β-glucans from oats reduces cholesterol levels in hypercholesterolemic individuals and therefore reduces the risk of heart disease. The recommended minimum dose is 3 g β-glucan per day. In this study, the diet contributed with the double amount, 6 g/day.

The multivariate analysis showed that there was a positive association between all SCFAs and oats, and a negative association between SCFAs and wheat. Wheat correlated to a greater extent with more gastrointestinal symptoms and impaired subjective health. Thus, constipation was worsened with wheat, which might be expected with a lower fiber intake, which is not controversial per se, and to a certain extent indigestion was more correlated to wheat than to oats. The impaired subjective health, including daily functions (SHS2), disease-related worries (SHS3), and general well-being (SHS4), which was more associated with wheat than the fiber-rich oats, was perhaps more unforeseen, but might be related to the increasing interest on the effects of the microbiota on circulating SCFAs and the brain.

It may be questioned why the relapse rate with the 2 diet groups was the same, although the fecal concentration of butyric acid increased with oats, a deterioration of gastrointestinal symptoms was prevented, and subjective health maintained. The reason for this is not known, but it may be speculated whether the butyric acid was too low to prevent relapses. It would be interesting to investigate oats with a higher proportion of β-glucans, which has been processed to contain dietary fiber polysaccharides with different solubility and molecular weights, and to analyze serum SCFAs more completely. It is important to carry out more dietary interventions to find more food items that can be used in the therapy of UC.

## CONCLUSIONS

Oat bran, contributing with 6 g β-glucan per day, given to patients with UC increased the butyrate concentration after 24 weeks, in contrast to the control diet consisting of wheat. There was no deterioration of gastrointestinal symptoms and the subjective health was maintained, which contrasted with the control group eating a low fiber diet containing wheat. However, the number of relapses was the same as with the control group eating wheat.

## Supplementary Material

otaa005_suppl_Supplementary_Table_1

## Notes

Conflicts of Interest and Source of Funding: The study was supported by Vetenskapsrådet (grant no 521-2004-5805) and Albert Påhlssons stiftelse. Lantmännen supported the study with products. The authors have no conflicts of interest to disclose.
