# Supplementary material for: Oat Bran Increased Fecal Butyrate and Prevented Gastrointestinal Symptoms in Patients With Quiescent Ulcerative Colitis—Randomized Controlled Trial
Source: Crohns Colitis 360. 2020 Feb 13;2(1):otaa005. doi: 10.1093/crocol/otaa005 (PMC9802401; doi:10.1093/crocol/otaa005)
Supplement: otaa005_suppl_Supplementary_Table_1 [file otaa005_suppl_supplementary_table_1.docx]

**Supplementary Table 1.** Correlation (Spearman) of short-chain fatty acids (SCFAs) and blood sugar to gastrointestinal symptoms and subjective health in patients with ulcerative colitis after intake of bread containing oat bran (n=47) or wheat (n=47) at week 24, where r = 0.1 is considered as a week correlation and r = 0.6 as strong correlation

|  | Diarrhea | Obstipation | Abdominal  pain | Reflux | Disease related worries /SHS 3 | General wellbeing /SHS 4 | LDL | Compliance |
| --- | --- | --- | --- | --- | --- | --- | --- | --- |
| Total SCFA^1^ | ns | -0.183 | ns | ns | ns | ns | ns | ns |
| Acetic acid^1^ | ns | -0.189 | ns | ns | -0.189 | -0.185 | ns | ns |
| Propionic acid^1^ | 0.177 | ns | ns | ns | ns | ns | -0.186 | ns |
| Butyric acid^1^ | ns | ns | ns | ns | -0.175 | ns | ns | ns |
| Valeric acid^1^ | ns | ns | ns | ns | ns | ns | ns | -0.206 |
| Isovaleric acid^1^ | ns | ns | ns | 0.181 | ns | 0.204 | ns | ns |
| Heptanoic acid^1^ | ns | 0.330 | 0.430 (*P* = 0.018) | ns | ns | ns | ns | ns |
| Blood sugar^1^ | ns | ns | ns | ns | ns | ns | 0.290 (*P* = 0.047) | -0.218 (*P* = 0.006) |

^1^ns, not significant
